# A Framework for Efficient N-Way Interaction Testing in Case/Control Studies With Categorical Data

**DOI:** 10.1109/OJEMB.2021.3100416

**Published:** 2021-07-27

**Authors:** Aristos Aristodimou, Athos Antoniades, Efthimios Dardiotis, Eleni M. Loizidou, George M. Spyrou, Christina Votsi, Kyproula Christodoulou, Marios Pantzaris, Nikolaos Grigoriadis, Georgios M. Hadjigeorgiou, Theodoros Kyriakides, Constantinos S. Pattichi

**Affiliations:** Department of Computer ScienceUniversity of Cyprus 54557 Nicosia 1678 Cyprus; Stremble Ventures Ltd. Limassol 59 4042 Cyprus; Department of Neurology, Faculty of MedicineUniversity of Thessaly 37786 Volos 38221 Greece; Department of Hygiene and EpidemiologyUniversity of Ioannina 37796 Ioannina 451 10 Greece; Institute for BioinnovationBiomedical Sciences Research Center Alexander Fleming, 54573 Athens, 16672 Greece; Bioinformatics Department and Cyprus School of Molecular MedicineCyprus Institute of Neurology and Genetics 87198 Nicosia 2371 Cyprus; Neurogenetics Department and Cyprus School of Molecular MedicineCyprus Institute of Neurology and Genetics 87198 Nicosia 2371 Cyprus; Department of Neurology and Cyprus School of Molecular MedicineCyprus Institute of Neurology and Genetics 87198 Nicosia 2371 Cyprus; Department of Neurology IIAristotle University of Thessaloniki 37782 Thessaloniki 541 24 Greece; Medical SchoolUniversity of Cyprus 54557 Nicosia 1678 Cyprus; Department of Basic and Clinical SciencesMedical School University of Nicosia 121343 Nicosia 1678 Cyprus; Department of Computer ScienceUniversity of Cyprus 54557 Nicosia 1678 Cyprus; Biomedical Engineering Research CentreUniversity of Cyprus 54557 Nicosia 1678 Cyprus

**Keywords:** Clustering, Epistasis, Feature Selection, Interaction Testing, Machine Learning

## Abstract

*Goal:* Most common diseases are influenced by multiple gene interactions and interactions with the environment. Performing an exhaustive search to identify such interactions is computationally expensive and needs to address the multiple testing problem. A four-step framework is proposed for the efficient identification of n-Way interactions. *Methods:* The framework was applied on a Multiple Sclerosis dataset with 725 subjects and 147 tagging SNPs. The first two steps of the framework are quality control and feature selection. The next step uses clustering and binary encodes the features. The final step performs the n-Way interaction testing. *Results:* The feature space was reduced to 7 SNPs and using the proposed binary encoding, more 2-SNP and 3-SNP interactions were identified compared to using the initial encoding. *Conclusions:* The framework selects informative features and with the proposed binary encoding it is able to identify more n-way interactions by increasing the power of the statistical analysis.

## I. Introduction

The introduction of affordable high throughput genotyping technologies allows the assay of millions of genetic polymorphisms per subject across the whole genome. This has led to genome wide association studies (GWAS) that try to identify genetic variations associated with diseases. Traditional genetic analyses focus on single locus associations, but most common diseases are influenced by multiple gene interactions and interactions with the environment [1].

Gene interaction is known in biology as epistasis, which is the result of physical interactions among biomolecules within gene regulatory networks and biochemical pathways in an individual, such that the effect of a gene on a phenotype is dependent on one or more other genes [2]. There is also the term of statistical epistasis, which is defined as the deviation from additivity in a statistical model, summarizing the relationship between multi-loci genotypes and phenotypic variations in a population [2].

Various methods have been proposed for interaction testing such as statistical [3], information theory [4], [5] and machine learning [6], [7] based methods. The statistical and information theory methods, usually provide a measure for identifying an interaction, but one needs to perform an exhaustive search on the available variables. To test for all possible n-Way interactions, one would need $O(k^n)$ tests, where $k$ is the total number of variables. This increases the computational complexity of the analysis. Additionally, in statistical tests that compute a p-value, if Bonferroni correction is used to address the problem of multiple testing, many true positive interactions will be missed, because of the conservative nature of this adjustment and the large number of tests performed [7].

Machine learning algorithms have also been proposed for identifying interacting factors. The Multifactor Dimensionality Reduction (MDR) algorithm [8], has been used in many studies [9]–[12]. This method is model free and reduces the dimensionality of tested factors by labeling each pattern as “high risk” or “low risk” if the ratio of cases:controls is above a threshold. Then using these labels and Cross Validation (CV), the model is evaluated. Random Forest (RF) [13] based methods have also been proposed for interaction testing [14], [15], but have not been as widely used as the MDR based methods.

Machine learning algorithms for pattern recognition can also be used for identifying interesting patterns associated with an outcome. The features involved in these patterns can then be used to test if interactions also exist. This could involve association rule mining alogorithms [16]–[19] that try to identify frequent patterns in the data, and clustering algorithms [20]–[22] that try to cluster instances in different groups based on their features similarity. In clustering algorithms, one can see the most frequent values of each cluster and the class they represent and see if there are any interesting patterns that are different between the classes and focus on those [23].

Applying machine learning algorithms directly on high dimensional data can be computationally demanding and can result in models that are hard to interpret [24]. Additionally in the case that the number of features is much larger than the number of instances, a common case in biomedical data, it is hard to identify the true signals from noise and there is also the risk of overfitting [25]. To reduce the search space before performing interaction testing and to also alleviate the issues aforementioned for machine learning methods, feature selection can be used [24], [26]. Feature selection, tries to identify the smallest subset of the available features that can best explain the response variable. Various feature selection algorithms have been used in genetic data such as Relief-F [27], the Recursive Feature Elimination Support Vector Machine (RFE-SVM) [28], and various penalized regression methods [29].

In this paper we propose a machine learning framework for discovering n-Way interactions associated to disease. The goal is to use machine learning to reduce the dimensionality of the data and therefore limit the multiple testing problem and the complexity of the analysis. Then subjects are clustered based on their underlying genetic profile across multiple genetic loci. Finally, using the most frequent genotypes of the cluster of interest, the genotypes are converted to binary to reduce the degrees of freedom of the interaction tests that will be performed and increase the statistical power. The proposed framework can be applied to categorical data to identify interactions between genetic, environmental and phenotypic features that are associated with the susceptibility of a disease. In this paper it is evaluated on real data from a Multiple Sclerosis study for the identification of 2-SNP and 3-SNP interactions.

## II. Materials and Methods

The dataset used in the experiments is presented along with the evaluation methodology of the proposed framework. For each step of the proposed framework, the algorithms used are extensively described.

### A. Dataset

The distribution of the subjects in the dataset used in this study is shown on Table 1. It consists of 389 Multiple Sclerosis (MS) patients and 336 controls from 3 MS centers; The Cyprus Institute of Neurology and Genetics in Cyprus, the University Hospital of Larissa, and the AHEPA Hospital of Aristotle University. The stydy was reviewed by the Cyprus National Ethics Committee EEBK/E$\Pi$/2010/02 on the 13th of April 2010.

**TABLE I:** Subjects Distribution

	Males	Females	Total
Cases	138	251	389
Controls	102	234	336
Total	240	485	725

All patients are of Greek origin with 240 of them being males and 485 of them being females. The dataset has 147 tagging Single Nucleotide Polymorphisms (SNPs) across 9 genes encoding for P-selectin (SELP), integrins (ITGA4, ITGB1, and ITGB7), adhesion molecules (ICAM1, VCAM1, and MADCAM1), fibronectin 1 (FN1), and osteopontin (SPP1) [30]. The genotypes of the SNPs were assigned with the value of 1 for homozygous minor alleles (aa), the value of 2 for heterozygous alleles (aB/Ba) and the value of 3 for homozygous major alleles (BB). Missing values were represented by the value of 0.

### B. Proposed Framework

The proposed framework is comprised of four steps. The first step is on quality control to remove any erroneous data. The next two steps use machine learning to perform feature selection and to cluster subjects based on their similarities. The final step uses the processed data of the previous steps to convert each feature into a binary variable before performing an n-Way interaction testing. Essentially, the last step reduces the dimensionality of each feature and hence the degrees of freedom of the statistical test that will be performed. This, increases the statistical power of the analysis. The proposed framework can be applied on categorical data and hence any continuous features that need to be included in the analysis should be discretized. The features can be of any type (e.g. genetic, environmental, phenotypic), which means that the analysis is not limited in the identification of only interacting genetic data, but also the interactions between genetic and environmental/phenotypic data.

#### 1) Quality Control

The purpose of the first step is to perform data cleaning to remove data of low quality that could negatively affect the analysis. In this step, one can use any of the well established methods for quality control in genetic, phenotypic and environmental data. Some of these are the Hardy-Weinberg Equilibrium (HWE) test, the minimum Minor Allele Frequency (MAF) filter and the percentage of missing values filter.

#### 2) Feature Selection

Once data have been cleaned, supervised feature selection is used. Since the ultimate goal is to identify interactions, it is important to use an algorithm that can select such interacting features. This means that it is better to avoid using feature selection algorithms that perform univariate analysis, since this can exclude interacting terms that have low main effects. Another important characteristic of the feature selection algorithm that will be used, is to use a similar modeling function as the interaction testing method. For example, if the interaction testing method is model-free, then it is best if the feature selection algorithm is also model-free, otherwise important features could be removed.

#### 3) Clustering

The next step is to cluster subjects based on their genotype. The similarity between data is usually calculated using the Hamming distance when they are categorical. In this framework, it is of interest to group subjects that have similar genotypic/environmental/phenotypic patterns together and use those to help perform a more targetted interaction testing. For this reason, features are handled as nominal categorical variables in the clustering procedure, since we do not want to consider an order in the values of each feature. Once clustering is performed, each resulted cluster will have a different number of cases and controls. If the majority of the subjects in that cluster are Cases then the cluster will be labeled as a Cases cluster otherwise as a Controls cluster. Based on these requirements, it is important to use a clustering algorithm that can handle nominal categorical data such as the NC-SOM [31] and k-modes [32].

Finally, to select the appropriate number of clusters and to see if the resulting clustering is of interest, the clusters should be evaluated. This can be tested using Pearson's $\chi ^2$ test on the number of cases against the number of controls in each cluster, as was previously proposed [23]. Using this test, one can see if the distribution of cases and controls in the produced clusters deviates from the expected and hence indicates that the patterns in the clusters could be used to separate the two classes.

#### 4) N-Way Interaction Testing

In the last step, given that the clustering had statistically significant results, the features will be encoded to binary variables. This is done by initially identifying clusters that are of interest, based on the ratio of cases and controls, the size of the clusters and how distinctive the patterns of a cluster of a certain label are from the patterns of clusters of other labels.

Once a cluster of interest is identified, the features’ values are encoded into binary, based on the most frequent value the subjects of that cluster have for that feature. For example, if we had SNPs and for a SNP, the subjects in the cluster of interest mainly have heterozygous alleles then aB and Ba will be encoded to the value of 1 and the homozygous minor alleles (aa) and homozygous major alleles (BB) will be encoded to 0. Then n-Way interaction testing is applied to all subjects using the new encoding.

This allows performing targeted interaction testing, since the clustering already identified the values that are common in the cluster of a certain class. Hence, we are now investigating if these patterns also have an interacting effect. Additionally, since we have reduced the dimensionality of each feature to binary, the statistical power of the analysis is increased, which allows identifying such interactions with smaller sample sizes as well.

### C. Evaluation Methodology

This section outlines the methods used in this study in each step of the proposed framework. Additionally the methodology followed for evaluating the effectiveness of the framework is described.

#### 1) Quality Control

For data cleaning, two data characteristics were used. The first one was the percentage of missing data of each SNP, and the second one the MAF. Specifically, any SNPs with more than 5% missing values or with an MAF less than 5% were removed from the dataset.

#### 2) Feature Selection

For features selection, Random Forest (RF) [13] was used for finding the importance of the SNPs regarding their ability of differentiating between cases and controls. RF is a collection of many de-correlated trees and to decide in which class a subject belongs to, each tree votes and the class with the majority of votes is selected. The algorithm creates each tree using bootstrapping hence at each tree some subjects will be used more than once, whereas others may not. The subjects not used are called the out of bag (OOB) samples and are used for calculating the misclassification error.

In this work, the implementation from the R package randomForest was used [33] using 50 trees and the importance of SNPs was measured by the Mean Decrease in Gini.

#### 3) Clustering

For clustering, the NC-SOM [31] for nominal categorical data was used, following the methodology described in [23] for selecting the appropriate map size. SOMs have the ability of assigning subjects with similar genetic data to neighboring clusters. Hence clusters that are close together tend to have subjects that are similar to each other compared to subjects in clusters in further locations. The distance between a cluster and a subject is calculated using the Hamming distance, which is more appropriate for nominal categorical data. Additionally the weights of each cluster, which represent the center of the cluster, are the modes of each cluster and hence one can see the most frequent values of each SNP for that cluster. SNPs are encoded to the genotype level with the values $\lbrace 0,1,2,3\rbrace$ for $\lbrace missing,aa,aB/Ba,BB\rbrace$ respectively.

Different map sizes are evaluated on the input dataset using a stratified 2-fold Cross Validation (CV). Due to the initial randomization of the weights of SOM, the 10-fold cross validation procedure is repeated 10 times. To select the appropriate map size for SOM, the test was repeated on different map sizes for 1000 epochs. The initial neighbourhood radius is set to half the map's edge size. The $\chi ^2$ test is used for evaluating the clustering on both training and testing data, by applying it on the contingency table that has the number of cases and controls of each cluster. The map size with the lowest p-value is selected and if it is statistically significant $(p-value< =0.05)$ the clustering is repeated with all data and the analysis proceeds to the next step of the framework.

#### 4) n-SNP Interaction Testing

For interaction testing, two methods were used to test if the proposed framework behaves similarly in different methods. The first method is the logistic regression, which is the most natural way to test for statistical interaction on the log odds scale [34] and is widely used for interaction testing. The second method is based on a $\chi ^2$ interaction testing approach [35]. The latter method is better suited for use on larger datasets because it is less computationally demanding but it tends to quantify the interaction effect lower than the logistic regression [36], making it a stricter measure.

For the logistic regression, the model used for 2-SNP interaction testing is shown below

\begin{equation*}
ln\left(\frac{p}{1-p}\right) = \beta + \beta _A x_A + \beta _B x_B + \beta _{AB}x_Ax_B \tag{1}
\end{equation*}

in which $x_A$ and $x_B$ are the two SNPs of interest at loci A and B respectively, $\beta _A$ and $\beta _B$ are regression coefficients that represent the main effects of exposures at $A$ and $B$, and the coefficient $\beta _{AB}$ represents an interaction term [34]. Similarly the model can be extended for 3-SNP interactions as shown below

\begin{align*}
ln\left(\frac{p}{1-p}\right) &= \beta + \beta _A x_A + \beta _B x_B + \beta _C x_C + \beta _{AB}x_Ax_B \\
&\qquad + \beta _{AC}x_Ax_C + \beta _{BC}x_Bx_C + \beta _{ABC}x_Ax_Bx_C \tag{2}
\end{align*}

The model for 2-SNP interaction testing using the $\chi ^2$ method is shown below

\begin{equation*}
\chi ^2_{A*B} = \chi ^2_{A+B} - \chi ^2_A - \chi ^2_B \tag{3}
\end{equation*}

where $\chi ^2_{A+B}$ is the omnibus effect of SNPs A and B, $\chi ^2_A$ is the main effect of SNP A and $\chi ^2_B$ is the main effect of SNP B. To calculate the degrees of freedom, the following model is used

\begin{equation*}
DF_{A*B} = DF_{A+B} - DF_A - DF_B \tag{4}
\end{equation*}

where $DF_{A+B}$ is the degrees of freedom of the $\chi ^2$ test performed on the omnibus effect of SNPs A and B and similarly $DF_A$, $DF_B$ for the main effect of SNPs A and B respectively. To test for 3-SNP interactions the model is extended as shown below

\begin{equation*}
\chi ^2_{A*B*C} \!=\! \chi ^2_{A+B+C} \!-\! \chi ^2_{A*B} \!-\! \chi ^2_{B*C} \!-\! \chi ^2_{A*C} \!-\! \chi ^2_A\!-\!\chi ^2_B -\chi ^2_C \tag{5}
\end{equation*}

and its degrees of freedom can be calculated using the following model

\begin{align*}
DF_{A*B*C} &= DF_{A+B+C} - DF_{A*B} - DF_{B*C} - DF_{A*C}\\
&\qquad - DF_A - DF_B - DF_C \tag{6}
\end{align*}

Interaction testing was performed on both the binary version of the genotypes and on the initial encoding, to test if the proposed encoding helps with the task at hand. An exhaustive 2-SNP and 3-SNP interaction testing was performed on the selected SNPs. In each tested SNPs pair/triplet, the subjects that had a missing value in the SNPs of that pair/triplet, were removed from the analysis. An interaction was considered statistically significant if it had a p-value less than 0.05. To address the multiple testing problem, permutation analysis was performed to adjust the p-values using 10 000 permutations.

## III. Results

**Fig. 1.**
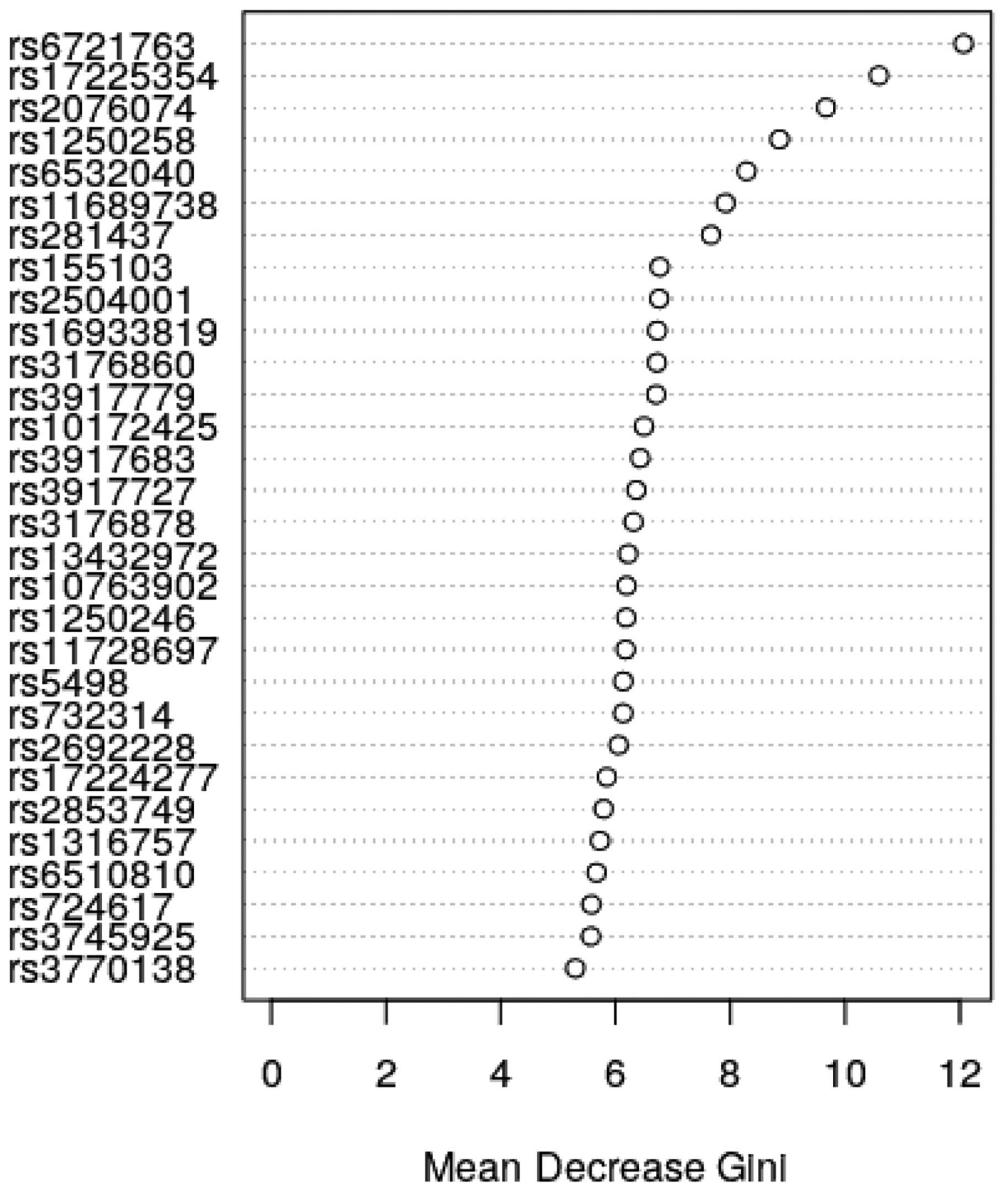
The importance of the top 30 SNPs from the Random Forest analysis using the mean decrease in Gini.

During quality control, 80 SNPs failed to pass the missing data threshold and from the remaining ones two more failed to pass the MAF threshold. Hence the Random Forest had 65 SNPs to analyze. The SNPs importance plot is shown in Fig. 1.

Based on the mean decrease in Gini, the top 7 SNPs of Fig. 1 were selected.

**Fig. 2.**
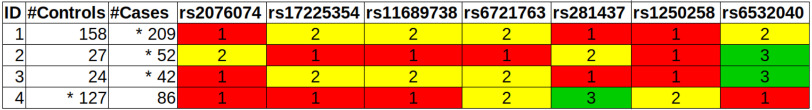
Each row represents a cluster of the SOM. The first two columns indicate the number of cases and controls each cluster has and an asterisk indicates if the majority of subjects is cases or controls. The rest of the columns show the most frequent genotype of each SNP for the subjects that belong to it.

After testing different map sizes for the SOM using cross validation, the final topology was selected to be a 2x2 map. The clustering with all subjects using the selected SNPs from Random Forest had a p-value of 0.00 002, indicating that the distribution of cases and controls in the clusters was deviating from the expected. The final clusters are shown in Fig. 2.

Each row represents a cluster and has its identification number (ID) in the first column and the number of cases and controls in the next two columns. An asterisk indicates the majority class of a cluster. Then for each SNP the most frequent genotype of the subjects that belong to that cluster is shown. Red indicates homozygous minor alleles, yellow heterozygous alleles and green homozygous major alleles. As indicated on Fig. 2, the first three clusters (IDs 1-3), include a majority of cases in them and only the last cluster (ID 4) has a majority of controls. The encoding of the SNPs to binary, will be based on the controls cluster to evaluate if there are SNP interactions with those genotypes that can help separate cases from controls. For example, SNP rs281437 will have the value 1 for its homozygous major alleles and 0 for the rest.

The interaction testing results are shown in Table 2. Both logistic regression and the $\chi ^2$ method identified more 2-SNP and 3-SNP interactions using the proposed binary encoding compared to using the initial encoding. In both methods there was a single 2-SNP interaction that was only identified using the initial encoding, whereas using the binary encoding the logistic regression identified four more SNP interactions and the $\chi ^2$ method five more that were not identified using the initial encoding.

**TABLE II:** Statistically Significant n-SNP Interactions Per Method and SNP Encoding

	**Initial Encoding**	**Binary Encoding**	**Initial Encoding**	**Binary Encoding**
**2-SNP interactions**	**Logistic**		$\mathbf {\chi ^2}$	
rs17225354 (ITGA4) & rs6721763 (ITGA4)	✓		✓	
rs17225354 (ITGA4) & rs281437 (ICAM1)	✓	✓		✓
rs17225354 (ITGA4) & rs1250258 (FN1)		✓		✓
rs2076074 (SELP) & rs6721763 (ITGA4)		✓		✓
rs11689738 (ITGA4) & rs281437 (ICAM1)	✓	✓	✓	✓
rs11689738 (ITGA4) & rs6721763 (ITGA4)		✓		✓
**3-SNP interactions**	**Logistic**		$\mathbf {\chi ^2}$	
rs17225354 (ITGA4) & rs281437 (ICAM1) & rs1250258 (FN1)	✓	✓		
rs2076074 (SELP) & rs6721763 (ITGA4) & rs281437 (ICAM1)		✓		✓

## IV. Discussion

The aforementioned results indicate that the proposed framework is able to reduce the computational needs of the analysis by reducing the number of features to be analysed through quality control and feature selection. If one performed only the quality control step, which is standard in such analyses, s/he would end up with 65 SNPs. To test all 2-SNP and 3-SNP interactions with 10 000 permutations one would need to perform around 450 million tests, whereas with the proposed framework one needs to perform around 55 thousand tests on the selected 7 SNPs. The proposed framework also allows the identification of more 2-SNP and 3-SNP interactions by using a targeted binary encoding based on the most frequent values of the genotypes of the cluster of interest.

The selected 7 SNPs after the quality control and the feature selection step were also found to have an association with the case/control status of the subjects in a previous study [30]. This means that in the selected SNPs there are no SNPs with a low main effect and any possible interactions in this case are between SNPs that have high main effects. Random Forests can include SNPs that have low main effects if they are interacting with SNPs with high main effects, but if the interacting SNPs are all with low main effects it is possible that they will be missed [6], hence such interactions might have been missed in the results.

As expected, since the $\chi ^2$ method is more strict, it identified fewer 2-SNP and 3-SNP interactions as statistically significant compared to the logistic regression. But it is clear that both methods benefited by the proposed encoding which allowed them to identify more interactions that were statistically significant. There are two reasons for being able to identify more n-SNP interactions using the proposed encoding. Initially, with binary encoding, the degrees of freedom of the statistical tests are reduced and hence the power of the analysis is increased. For example, in the $\chi ^2$ method, using the initial encoding in the 2-SNP interaction test we ended up with four degrees of freedom, whereas using the binary encoding with only one. Similarly, for the 3-SNP interaction test, we dropped from 8 degrees of freedom to one. The second reason being that the encoding enforces a more targeted analysis on the possible interacting SNP values that can be seen from the clustering step, which shows the SNP genotypes that are more frequent in the cluster of interest versus the remaining, which in this case is the controls cluster versus the cases clusters.

The identified interacting SNPs have been associated with MS risk and genes that encode proteins with crucial functions for MS. The intronic polymorphism (intron 17–18) ITGA4 rs6721763, located 929 bp away from the splicing site of exon 18 of ITGA4 gene, has been previously associated the MSSS in a dose-dependent manner and also with the MS risk, while the intronic rs17225354 has been associated with MS risk [30], [37]. This is of particular interest, as at least one of these two variants is included at all the 2-SNP and 3-SNP interactions that have been identified with the current method. The ITGA4 gene encodes the $\alpha 4$ sub-unit of the $\alpha 4\beta 1$ integrin, which is implicated to the adhesion and migration procedures of leukocytes through the blood-brain barrier and also ITGA4 is among therapeutic targets with the monoclonal antibody natalizumab [38], [39]. The remaining of the SNPs included in the 2-SNP and 3-SNP interactions that have been identified with the current method, are located also in genes encoding proteins with crucial functions for MS. For example the FN1 is an extracellular matrix glycoprotein that interacts with integrins, while the ICAM1 which is an adhesion molecule expressed in the CNS extracellular space and SELP play a pivotal role for the interaction between activated endothelial cells or platelets and leukocytes [38], [40], [41]. In view of the former consideration, it is possible that each of the SNPs confer some susceptibility to MS, while their interactions may have robust biological connections leading to the disease.

There are two aspects of the framework to be evaluated. The first is whether its initial 2 steps can help reduce the search space in an informed way which will enable interaction testing that would otherwise be computationally expensive to perform. The second is whether the proposed encoding can help identify more n-Way interactions that are statistically significant. We can conclude that the proposed framework works, since it was able to limit the number of tests performed and it was able to identify more 2-SNP and 3-SNP interactions that were statistically significant even after permutation adjusting for multiple testing. The fact that most of the statistically significant identified interactions came up in both methods, indicates that at least those interactions adhere to the statistical assumptions of both methods that further supports that the results obtained are not due to random effects and that the proposed encoding can work with different interaction testing methods. However, as to whether the results of the two methods are true-positive, that would require an independent study to be performed to validate the findings.

## V. Conclusion

A framework for the efficient identification of n-Way interactions has been proposed. The framework removes any erroneous data in its first step and then uses feature selection to reduce the dimensionality of the search space and select the most informative features. This, reduces the statistical tests that will be performed and hence the multiple testing problem. Additionally, it reduces the computational complexity of the analysis to be performed and the probability of overfitting. Therefore, the first two steps of the framework focus on providing high quality and informative features.

In the clustering step, subjects are grouped based on their similarity in their genotypes and each cluster is labeled based on the majority class of the subjects. This enables the visualization of the most frequent values of the subjects in each cluster and the phenotype they represent. Then a cluster of interest is selected and its values are used to convert the genotypes to binary variables. This enables a targeted interaction testing analysis and additionally increases the power of the statistical analysis due to the reduction of the degrees of freedom of the tests. As has been shown in the results, using the binary encoding more 2-SNP and 3-SNP interactions were identified, which further supports the hypothesis that the proposed framework helps identifying more interactions.

Additionally, due to the cluster based binary encoding, the framework can also promote personalised medicine. For example, one could identify different n-Way interactions for each cluster of interest and hence adjust the treatment of a patient based on his/her genotypic profile.
